# Disease-Alleviating Effects of Peroral Activated Charcoal Treatment in Acute Murine Campylobacteriosis

**DOI:** 10.3390/microorganisms9071424

**Published:** 2021-06-30

**Authors:** Stefan Bereswill, Soraya Mousavi, Dennis Weschka, Markus M. Heimesaat

**Affiliations:** Gastrointestinal Microbiology Research Group, Institute of Microbiology, Infectious Diseases and Immunology, Charité-Universitätsmedizin Berlin, Corporate Member of Freie Universität Berlin, Humboldt-Universität zu Berlin and Berlin Institute of Health, 12203 Berlin, Germany; stefan.bereswill@charite.de (S.B.); soraya.mousavi@charite.de (S.M.); dennis.weschka@charite.de (D.W.)

**Keywords:** activated charcoal, enteropathogenic infection, *Campylobacter jejuni*, immune-modulatory effects, microbiota-depleted IL-10^−/−^ mice, campylobacteriosis model, host–pathogen interaction, preclinical intervention study, natural antibiotics-independent compounds

## Abstract

Foodborne *Campylobacter jejuni* infections are on the rise and responsible for worldwide serious health issues. Increasing resistance of *C. jejuni* strains against antimicrobial treatments, necessitates antibiotics-independent treatment options for acute campylobacteriosis. Activated charcoal (AC) constitutes a long-known and safe compound for the treatment of bacterial enteritis. In this preclinical intervention study, we addressed potential anti-pathogenic and immune-modulatory effects of AC during acute experimental campylobacteriosis. Therefore, microbiota-depleted IL-10^−/−^ mice were infected with *C. jejuni* by gavage and challenged with either AC or placebo via the drinking water starting on day 2 post-infection. On day 6 post-infection, AC as compared to placebo-treated mice did not only harbor lower intestinal pathogen loads but also presented with alleviated *C. jejuni*-induced clinical signs such as diarrhea and wasting symptoms. The improved clinical outcome of AC-treated mice was accompanied by less colonic epithelial cell apoptosis and reduced pro-inflammatory immune responses in the intestinal tract. Notably, AC treatment did not only alleviate intestinal, but also extra-intestinal and systemic immune responses as indicated by dampened pro-inflammatory mediator secretion. Given the anti-pathogenic and immune-modulatory properties of AC in this study, a short-term application of this non-toxic drug constitutes a promising antibiotics-independent option for the treatment of human campylobacteriosis.

## 1. Introduction

Foodborne campylobacteriosis due to infections with the Gram-negative bacterial enteropathogen *Campylobacter jejuni* cause significant burdens to the public health systems all around the globe [1,2]. Since prevalence rates of human *C. jejuni* infections are progressively increasing worldwide, this infectious disease poses a global health issue which can only be solved by so called “One Health” approaches combining research efforts in human and veterinary medicine with governmental interventions and consumers’ protection [3,4,5]. The intestinal tract of avian vertebrates constitutes the major reservoir for the pathogen where *C. jejuni* bacteria behave as commensal residents and usually do not cause clinical signs in the colonized host [5]. In consequence, *C. jejuni* is transferred to humans from avian livestock via contamination within the food chain. It is known since decades that infected patients present with diarrhea, abdominal pain, vomiting, headache, fever, diarrhea, and bloody stools [6,7]. In otherwise healthy subjects, *C. jejuni*-induced disease is usually self-limiting and resolves within 10 to 14 days post-infection (p.i.) [8]. Antibiotic treatment, however, is reserved for severely diseased hospitalized cases including immune-compromised patients who are at risk for systemic complications [4]. Therefore, only symptomatic treatment consisting of fluid replacement and application of analgetic compounds is indicated in the majority of cases. One should take into consideration, however, that even the self-limiting cases result in a significant socioeconomic burden, which is accelerated by post-infectious diseases affecting the central nervous system, the intestinal tract, or the joints. These rare autoimmune morbidities include Guillain-Barré and Miller Fisher syndromes, Bickerstaff encephalitis, irritable bowel syndrome, celiac disease, inflammatory bowel diseases, and reactive arthritis [3,9,10,11,12].

The pathogenesis of human campylobacteriosis is multifactorial. In the absence of an exotoxin produced by all *C. jejuni* strains, the clinical manifestation of infectious enteritis is caused by endotoxins including lipo-oligosaccharide (LOS), a truncated form of lipo-polysaccharide (LPS) which triggers inflammatory responses by the intestinal innate immune system [13,14]. Cellular infiltrates within the infected intestinal mucosa and lamina propria consisting of leukocytes, granulocytes, monocytes, and macrophages as well as apoptotic intestinal epithelial cells are hallmarks of campylobacteriosis from the pathologists’ perspective [15]. Following oral ingestion and successful gastroduodenal passage, the highly motile *C. jejuni* bacteria move through the viscous mucous barrier and penetrate intestinal epithelial cells upon attachment, invasion and transmigration [16]. In the sub-epithelial layer *C. jejuni* induce a recruitment of macrophages, monocytes, granulocytes, and dendritic cells to the site of infection [17,18]. These innate immune cells are attracted and become activated by *C. jejuni* surface LOS. The resulting intestinal inflammation causes apoptosis and cell death by immune mediators including reactive oxygen species (ROS) and pro-inflammatory cytokines [19]. In consequence, epithelial barrier destruction and disturbance of the sodium equilibrium lead to a leaky gut syndrome with malabsorption and diarrhea [20]. In severe and invasive campylobacteriosis cases, inflammatory responses may become overt in extra-intestinal sites and even systemically [3,6]. Furthermore, both, severity of *C. jejuni*-induced clinical symptoms as well as the risk for development of post-infectious collateral damages have been shown to be directly linked to the sialylation status of the LOS derived from the cell wall of the infecting *C. jejuni* strain [12].

In order to evaluate novel strategies for the treatment and prophylaxis of campylobacteriosis, we survey well-known non-toxic and natural molecules including probiotic compounds regarding their anti-pathogenic and immune-modulatory properties by applying microbiota-depleted IL-10^−/−^ mice as an experimental model for acute campylobacteriosis. Within a week p.i., these animals have been shown to develop acute enterocolitis characterized by bloody diarrhea and wasting symptoms and to display microscopic inflammatory features such as colonic epithelial apoptosis and cellular aggregates such as macrophages, monocytes, granulocytes, and lymphocytes infiltrating the infected intestinal mucosa and lamina propria [21]. In turn, pro-inflammatory mediators are released in order to limit the infection but by the expense of perpetuating the cellular damage to the host [22,23]. Intestinal as well as systemic inflammatory sequelae of *C. jejuni* infection have been shown to be due to innate and adaptive host immune responses that are induced by *C. jejuni*-LOS and mediated by Toll-like recptor-4 (TLR-4) signaling [14]. Thus, the microbiota-depleted IL-10^−/−^ mouse model resembles main immunopathological features of acute LOS-driven campylobacteriosis in humans [20,22,23]. Importantly, in these mice, sirolimus, a potent immune-suppressing drug acting on the mammalian target of rapamycin (mTOR), was shown to block both, *C. jejuni*-induced inflammation and bacterial accumulation in the intestinal tract [22]. The immune-modulatory approach for treating campylobacteriosis has been further proven valuable by previous preclinical placebo-controlled intervention studies. In particular, vitamin C [24], vitamin D [25], carvacrol [26], resveratrol [27,28], urolithin-A [29], essential oils derived from cardamom, clove and garlic [30,31,32], neuropeptides such as NAP and PACAP [33,34], probiotic compounds such as VSL#3 [35] and fecal microbiota transplantation [36,37] were shown to alleviate campylobacteriosis via immune-modulatory effects.

To investigate potentially novel and particularly antibiotics-independent treatment options for human campylobacteriosis in a preclinical setting applying a well-established murine *C. jejuni* infection and inflammation model, we searched for another natural substance lacking relevant adverse effects, which is pharmaceutically used in diarrheal diseases. In fact, activated charcoal (AC) appeared as a promising candidate fulfilling these prerequisites. After the preparation from natural carbon-containing plant materials and activation by extensive heating, AC has been used for the treatment of intoxications and infectious enteritis in traditional medicine for centuries [38]. Furthermore, clinical studies revealed evidence for disease-alleviating effects of AC in the treatment of intestinal morbidities such as irritable bowel syndrome and diarrhea caused by tumor chemotherapy, medications, or infectious enteric diseases [39]. Due to the large surface of the particles, which is further extended by pores, the main mode of action of AC is the binding and adsorption of toxins and infectious agents thereby protecting the human body from poisoning, intoxication, and enteropathogenic invasion [38]. Furthermore, anti-inflammatory effects of AC include binding and inactivation of pro-inflammatory cytokines and of bacterial endotoxins such as LOS [40]. However, direct antimicrobial or antibiotic activities of AC have not been reported so far. Hence, AC combines pathogen-inactivation and anti-inflammatory effects with lacking antibiotic activities. In consequence, these combinatory effects minimize the risk of both, resistance development by *C. jejuni* and intestinal dysbiosis.

In our actual preclinical and placebo-controlled intervention study, we therefore studied the disease-alleviating effects of peroral AC application to microbiota-depleted IL-10^−/−^ mice and surveyed the gastrointestinal pathogen loads, the macroscopic (i.e., clinical) and microscopic inflammatory (i.e., histopathological, apoptotic) outcomes, and pro-inflammatory immune responses in intestinal, in extra-intestinal and in systemic organs.

## 2. Materials and Methods

### 2.1. Ethical Statement

The clinical conditions of each mouse were monitored daily. The experiments in the murine infection and inflammation model were performed according to the European animal welfare guidelines (2010/63/EU) following approval by the commission for animal experiments (“Landesamt für Gesundheit und Soziales”, LaGeSo, Berlin, Germany; registration number G0104/19, approved on 15 July 2019). 

### 2.2. Microbiota-Depleted IL-10^−/−^ Mice

Breeding of IL-10^−/−^ C57BL/6j mice was maintained under specific pathogen-free conditions in the Forschungsinstitute für Experimentelle Medizin, Charité—Universitätsmedizin Berlin, Germany. Mice housed in cages with filter tops within an experimental semi-barrier under standard conditions had free access to autoclaved water and standard chow (ssniff R/M-H, V1534-300, Sniff, Soest, Germany). For microbiota depletion, 3-week-old female and male mice were challenged with an antibiotic cocktail as reported previously [41,42]. After transfer to sterile cages, mice received an antibiotic cocktail containing ampicillin plus sulbactam (1 g/L; Dr. Friedrich Eberth Arzneimittel, Ursensollen, Germany), vancomycin (500 mg/L; Hikma Pharmaceuticals, London, UK), ciprofloxacin (200 mg/L; Fresenius Kabi, Bad Homburg, Germany), imipenem (250 mg/L; Fresenius Kabi), and metronidazole (1 g/L; B. Braun, Melsungen, Germany) via the drinking water for eight weeks (ad libitum). Following quality control by culture and Real time-PCR to confirm absence of intestinal bacteria as described earlier [42], the microbiota-depleted mice were kept and handled under strict aseptic conditions. The animals received autoclaved tap water without antibiotics three days before *C. jejuni* infection to guarantee washout of antimicrobial substances.

### 2.3. Campylobacter Infection and Treatment Regimens

Live bacteria of *C. jejuni* strain 81–176 were obtained from thawed frozen stocks and grown on columbia agar (with 5% sheep blood) and selective karmali agar plates (both from Oxoid, Wesel, Germany). Age- and sex-matched microbiota-depleted IL-10^−/−^ mice (4-month-old littermates) were infected perorally with 10^9^ colony-forming units (CFU) of the pathogen on days 0 and 1 by gavage. Treatment with AC (purchased from Sigma-Aldrich, Munich, Germany) was performed from day 2 p.i. until the end of the observation period and applied to autoclaved tap water (final concentration of 10 g/L; *ad libitum*; daily dose of 2.5 g/kg body weight). The placebo control mice received autoclaved tap water only. In summary, the following cohorts of mice with respective numbers of animals (in parentheses) were surveyed in four independent experiments: (i) naive (non-infected, non-treated) mice (5/5/5/5); (ii) *C. jejuni*-infected, placebo-treated mice (7/7/7/6), (iii) *C. jejuni*-infected, AC-treated mice (6/5/5/5).

### 2.4. Gastrointestinal Pathogen Burdens

After oral infection by gavage, the numbers of live *C. jejuni* bacteria were determined in fecal samples daily and upon necropsy in intraluminal gastrointestinal samples that had been homogenized in sterile phosphate-buffered saline (PBS, Thermo Fisher Scientific, Waltham, MA, USA). *C. jejuni* was quantified by colony counting after growth of serial dilutions of intestinal samples on karmali agar (Oxoid, Wesel, Germany) for at least 48 h at 37 °C under microaerophilic conditions as described previously [42,43]. The detection limit of viable pathogens was 100 CFU per g (CFU/g) intraluminal gastrointestinal sample.

### 2.5. Monitoring of Clinical Conditions of Animals

Immediately before and after infection, we quantitatively surveyed the daily clinical outcome of mice by using a cumulative clinical score (maximum 12 points), addressing the clinical aspect of animals (i.e., wasting symptoms; 0: normal; 1: ruffled fur; 2: less locomotion; 3: isolation; 4: severely compromised locomotion, pre-final aspect), the occurrence of fecal blood (0: no blood; 2: microscopic detection of blood by the Guajac method using Haemoccult, Beckman Coulter/PCD, Krefeld, Germany; 4: macroscopic blood visible), and the stool consistency (0: formed feces; 2: pasty feces; 4: liquid feces), as described earlier [44].

### 2.6. Sampling Procedures

After sacrifice at day 6 p.i. by carbon dioxide asphyxiation, cardiac blood for cytokine measurements, and ex vivo biopsies from liver, kidneys, spleen, ileum, and colon as well as luminal samples from stomach, duodenum, ileum, and colon were removed under aseptic conditions. In parallel colonic samples were collected for subsequent microbiological, immunohistopathological and immunological analyses.

### 2.7. Histopathology

For histopathological analyses colonic ex vivo biopsies were immediately fixed in 5% formalin and embedded in paraffin. Sections (5 µm) were stained with hematoxylin and eosin (H&E), examined by light microscopy (100× magnification), and histopathology in the large intestines was quantitatively graded according to well-established histopathological scores [45]: Score 1, minimal inflammatory cell infiltrates in the mucosa with intact epithelium. Score 2, mild inflammatory cell infiltrates in the mucosa and submucosa with mild hyperplasia and mild goblet cell loss. Score 3, moderate inflammatory cell infiltrates in the mucosa with moderate goblet cell loss. Score 4, marked inflammatory cell infiltration into the mucosa and submucosa with marked goblet cell loss, multiple crypt abscesses, and crypt loss.

### 2.8. In Situ Immunohistochemistry

For in situ immunohistochemical analyses, colonic ex vivo biopsies were fixed in 5% formalin and embedded in paraffin as recently reported [46]. Apoptotic epithelial cells, macrophages and monocytes, T lymphocytes and B lymphocytes were counted in colonic paraffin sections (5 µm) stained with primary antibodies against cleaved caspase-3 (Asp175, Cell Signaling, Beverly, MA, USA, 1:200), F4/80 (No. 14-4801, clone BM8, eBioscience, San Diego, CA, USA, 1:50), CD3 (No. N1580, Dako, Glostrup, Denmark, 1:10), and B220 (No. 14-0452-81, eBioscience, San Diego, CA, USA, 1:200), respectively. An independent investigator counted numbers of specifically stained cells by light microscopy of blinded samples. The average number of respective positively stained cells in each sample was determined within at least six high power fields (HPF, 0.287 mm^2^, 400× magnification).

### 2.9. Pro-Inflammatory Mediators

Intestinal samples collected from the colon and ileum (longitudinally cut strips of approximately 1 cm^2^, washed in PBS) and ex vivo biopsies derived from mesenteric lymph nodes (MLN; 3 nodes), the liver (approximately 1 cm^3^), the kidney (one half after the longitudinal cut), the lung (one half), and the spleen (one third) were transferred to 24-flat-bottom well culture plates (Thermo Fisher Scientific, Waltham, MA, USA) containing 500 mL serum-free RPMI 1640 medium (Thermo Fisher Scientific, Waltham, MA, USA) supplemented with penicillin (100 µg/mL) and streptomycin (100 µg/mL; Biochrom, Berlin, Germany). After an 18-h incubation period at 37 °C, respective culture supernatants and serum samples were tested for interferon-γ (IFN-γ), interleukin-6 (IL-6), tumor necrosis factor-alpha (TNF-α), and monocyte chemoattractant protein-1 (MCP-1) by the Mouse Inflammation Cytometric Bead Assay (CBA; BD Biosciences, Heidelberg, Germany) on a BD FACSCanto II flow cytometer (BD Biosciences, Heidelberg, Germany). Nitric oxide was measured by the Griess reaction as described earlier [47].

### 2.10. Statistics

After pooling of data from four independent experiments, medians and significance levels were calculated using GraphPad Prism (version 8; San Diego, CA, USA). Normalization of data was surveyed by the Anderson–Darling test. The Mann–Whitney test was applied for pairwise comparisons of not normally distributed data. Multiple comparisons were performed using the one-way ANOVA with Tukey post-correction (for normally distributed data) and the Kruskal–Wallis test with Dunn’s post-correction (for not normally distributed data). Two-sided probability *p*-values of ≤0.05 were considered significant. Definite outliers were removed after being identified by the Grubb’s test (α = 0.001).

## 3. Results

### 3.1. Gastrointestinal Pathogen Numbers Following Treatment of Infected IL-10^−/−^ Mice with Activated Charcoal

**Figure 1:**
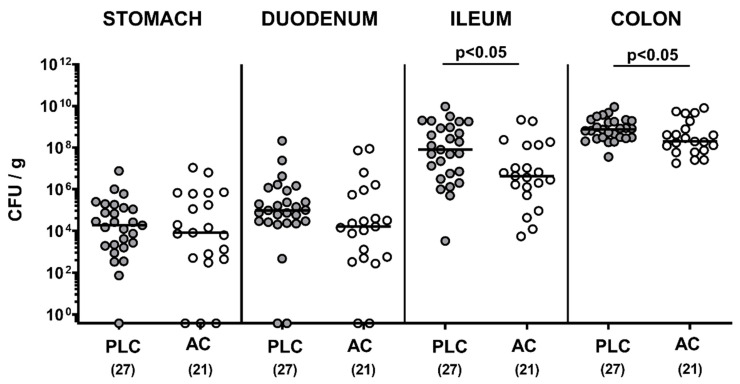
Gastrointestinal *C. jejuni* numbers following treatment of infected mice with activated charcoal. Microbiota-depleted IL-10^−/−^ mice were generated as described in methods and infected with *C. jejuni* strain 81–176 on day (d) 0 and d1 by gavage. Starting on d2 post-infection (p.i.), mice were perorally treated with either activated charcaol (AC; white circles) or placebo (PLC; gray circles) via the drinking water. The *C. jejuni* numbers in colony-forming units per gram (CFU/g) were determined in luminal samples of the gastrointestinal tract by culture. Samples were taken after sacrifice of mice on d6 p.i. Medians (black bars) and significance levels (*p*-values) were determined by the Mann–Whitney test using pooled data from four independent experiments. Total numbers of animals are shown in parentheses.

We first addressed whether oral treatment of *C. jejuni*-infected mice with AC affected pathogenic colonization of the gastrointestinal tract. Following peroral infection with 10^9^ viable bacterial cells, *C. jejuni* could stably establish in the intestinal tract of microbiota-depleted IL-10^−/−^ mice with high median numbers of 10^9^ CFU per gram large intestinal content (Figure 1). Our cultural analyses revealed that on day 6 p.i., AC-treated mice harbored approximately 0.5 and 2.0 log orders of magnitude lower median *C. jejuni* numbers in the colon and ileum as compared to placebo controls, respectively (*p* < 0.05), whereas in more proximal parts such as the duodenum and stomach, pathogen counts were comparable (not significant (n.s.); Figure 1). Hence, AC treatment resulted in lower intestinal pathogen loads of *C. jejuni*-infected IL-10^−/−^ mice.

### 3.2. Clinical Outcome Following Treatment of Infected Mice with Activated Charcoal

**Figure 2:**
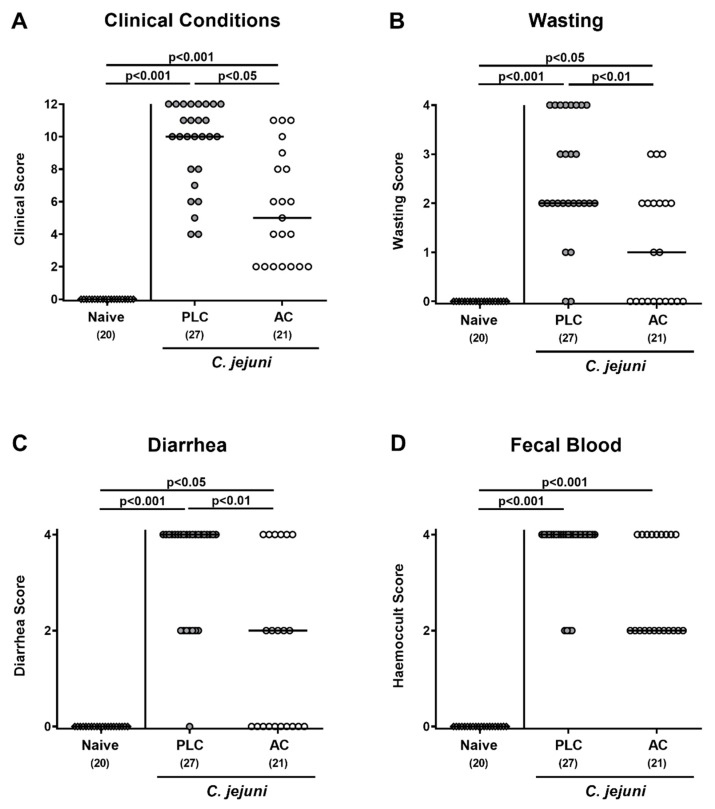
Clinical outcome following treatment of infected mice with activated charcoal. Microbiota-depleted IL-10^−/−^ mice were infected with *C. jejuni* strain 81–176 on day (d) 0 and d1 by gavage. Starting on d2 post-infection (p.i.), mice were perorally treated with either activated charcoal (AC; white circles) or placebo (PLC; gray circles) via the drinking water. (**A**) The overall clinical conditions of mice were quantitatively determined on d6 p.i. by using a cumulative clinical scoring system (see methods) assessing (**B**) wasting symptoms, (**C**) diarrhea, and (**D**) fecal blood. Data from naive mice serving as non-infected, untreated controls are indicated by white diamonds. Medians (black bars) and significance levels (*p*-values) were determined from pooled data of four independent experiments by the Kruskal–Wallis test and Dunn’s post-correction. The total numbers of analyzed mice are shown in parentheses.

We further surveyed the clinical signs of infected mice following AC treatment. On day 6 p.i., mice from the placebo group were suffering from acute campylobacteriosis characterized by bloody diarrhea and wasting symptoms (*p* < 0.001 versus naive; Figure 2), whereas AC-treated animals were less distinctly compromised as indicated by lower clinical scores for the overall clinical outcome, for wasting symptoms and for diarrhea when compared to placebo mice (*p* < 0.05–0.01; Figure 2A–C).

Of note, as early as day 2 p.i., AC as compared to placebo-treated mice were less distinctly suffering from *C. jejuni*-induced diarrhea given lower scores for stool consistency in the former versus the latter from day 2 until day 6 p.i. (*p* < 0.05–0.001; Supplementary Figure S1). Hence, AC treatment alleviated pathogen-induced clinical signs of acute murine campylobacteriosis.

### 3.3. Microscopic Inflammatory Changes in the Colon Following Treatment of Infected Mice with Activated Charcoal

**Figure 3:**
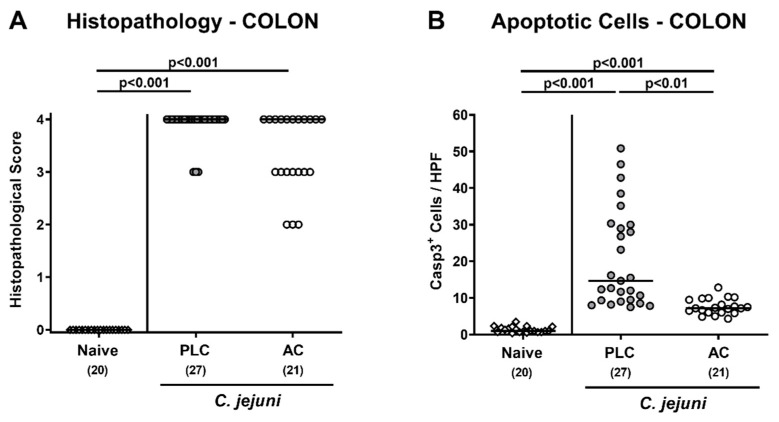
Microscopic inflammatory changes in the colon following treatment of infected mice with activated charcoal. Microbiota-depleted IL-10^−/−^ mice infected with *C. jejuni* strain 81–176 on day (d) 0 and d1 by gavage were perorally treated with either activated charcoal (AC; white circles) or placebo (PLC; gray circles) via the drinking water starting on d2 p.i. (**A**) Colonic histopathological changes were recorded in hematoxylin and eosin-stained colonic paraffin sections by using histopathological scores in samples collected on d6 p.i. In colonic samples taken on the same day the average numbers of (**B**) apoptotic colonic epithelial cells were microscopically determined from six high power fields (HPF, 400× magnification) in paraffin sections positive for cleaved caspase-3 (Casp3^+^). Data from naive mice serving as non-infected, untreated controls were included (white diamonds). Medians (black bars) and significance levels (*p*-values) were determined with the Kruskal–Wallis test and Dunn’s post-correction by using pooled data from four independent experiments. The total numbers of analyzed mice are shown in parentheses.

We next assessed the impact of AC treatment on microscopic inflammatory sequelae of *C. jejuni* infection. Therefore, we quantitated histopathological changes of the colonic mucosa by using histopathological scores on day 6 p.i. Upon *C. jejuni* infection pronounced histopathological changes within the large intestinal tract of mice from either treatment regimen could be assessed indicative for acute enterocolitis (*p* < 0.001 versus naive; Figure 3A). Of note, 52.4% of mice from the AC group displayed the highest histopathological score, whereas this was the case in 88.9% of the placebo control animals. Since apoptosis is used as a marker for the grading of intestinal inflammatory morbidities including acute campylobacteriosis [42], we furthermore quantitated apoptotic colonic epithelial cell numbers. In situ immunohistochemical analyses revealed that *C. jejuni* infection resulted in marked increases in cleaved caspase-3 positive colonic epithelial cell numbers (*p* < 0.001; Figure 3B). These increases were, however, far less pronounced in AC as compared to placebo-treated mice on day 6 p.i. (*p* < 0.01; Figure 3B). Hence, AC treatment alleviated pathogen-induced apoptosis in the colon of *C. jejuni*-infected mice.

### 3.4. Intestinal Pro-Inflammatory Immune Responses following Treatment of Infected Mice with Activated Charcoal

**Figure 4:**
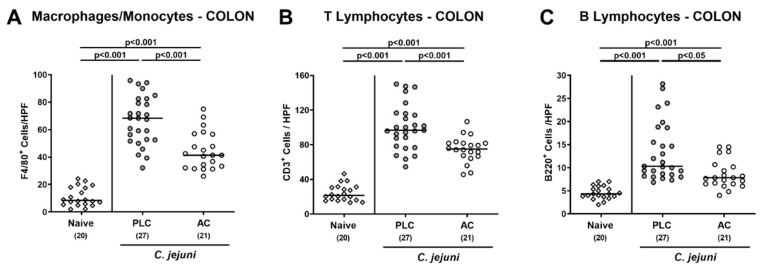
Immune cell responses in the colon following treatment of infected mice with activated charcoal. Microbiota-depleted IL-10^−/−^ mice infected with *C. jejuni* strain 81–176 on day (d) 0 and d1 by gavage were perorally treated with either activated charcoal (AC; white circles) or placebo (PLC; gray circles) via the drinking water starting on d2 post-infection (p.i.). On d6 p.i., the average numbers of (**A**) macrophages and monocytes (F4/80^+^), (**B**) T lymphocytes (CD3^+^), and (**C**) B lymphocytes (B220^+^) per mouse were determined in immunohistochemically stained colonic paraffin sections from six high power fields (HPF, 400× magnification). Data from naive mice serving as non-infected, untreated controls were included (white diamonds). Medians (black bars) and significance levels (*p*-values) were determined with the one-way ANOVA test with Tukey post-correction and the Kruskal–Wallis test and Dunn’s post-correction by using pooled data from four independent experiments. The total numbers of analyzed mice are shown in parentheses.

We next addressed potential immune-modulatory effects of AC treatment during acute murine campylobacteriosis. Therefore, we determined large intestinal numbers of distinct innate and adaptive immune cell populations following immunohistochemical staining of colonic paraffin sections. On day 6 p.i., increased numbers of macrophages and monocytes as well as of T- and B-lymphocytes could be assessed in the colonic mucosa and lamina propria (*p* < 0.001; Figure 4). These increases were, however, far less pronounced following AC as compared to placebo treatment (*p* < 0.05–0.001; Figure 4).

**Figure 5:**
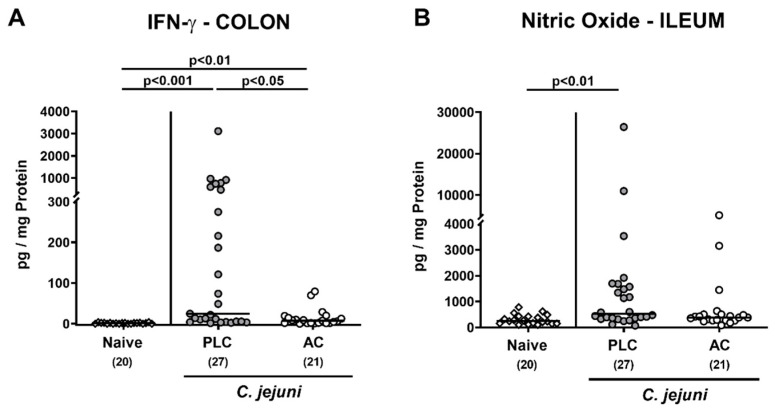
Intestinal pro-inflammatory mediator secretion following treatment of infected mice with activated charcoal. Microbiota-depleted IL-10^−/−^ mice were infected with *C. jejuni* strain 81–176 on day (d) 0 and d1 by gavage. Starting on d2 post-infection (p.i.), mice were perorally treated with either activated charcoal (AC; white circles) or placebo (PLC; gray circles) via the drinking water. On d6 p.i., (**A**) colonic IFN-γ and (**B**) ileal nitric oxide concentrations were measured in culture supernatants of respective ex vivo biopsies. Results from naive mice serving as non-infected, untreated controls were included for comparisons (white diamonds). Data pooled from four independent experiments were used to calculate medians (black bars) and significance levels (*p*-values) with the Kruskal–Wallis test and Dunn’s post-correction. The total numbers of analyzed mice are given in parentheses.

In addition, we measured pro-inflammatory mediators in distinct intestinal compartments. Upon AC treatment, lower IFN-γ concentrations could be assessed in the colon of *C. jejuni*-infected mice when compared to placebo controls (*p* < 0.05; Figure 5A). Moreover, in the ileum, increased nitric oxide concentrations were determined in placebo, but not AC-treated mice on day 6 p.i. (*p* < 0.01 versus naive; Figure 5B).

**Figure 6:**
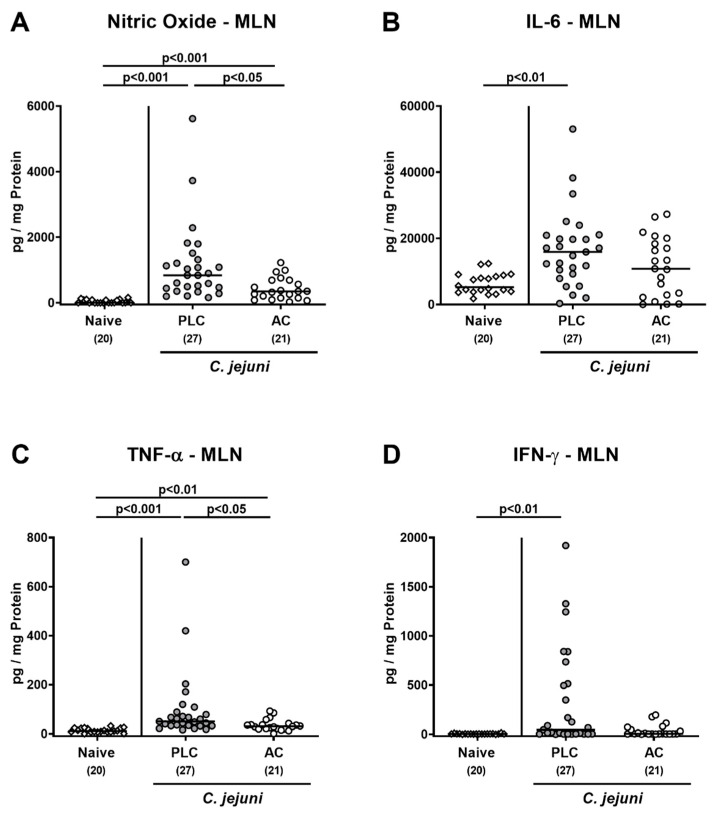
Pro-inflammatory mediator secretion in mesenteric lymph nodes following treatment of infected mice with activated charcoal. Microbiota-depleted IL-10^−/−^ mice infected with *C. jejuni* strain 81–176 on day (d) 0 and d1 by gavage were perorally treated with either activated charcoal (AC; white circles) or placebo (PLC; gray circles) via the drinking water starting on d2 post-infection (p.i.). On d6 p.i., (**A**) nitric oxide, (**B**) IL-6, (**C**) TNF-α, and (**D**) IFN-γ concentrations were measured in culture supernatants of ex vivo biopsies derived from mesenteric lymph nodes (MLN). Results from naive mice serving as non-infected, untreated controls were included for comparisons (white diamonds). Data pooled from four independent experiments were used to calculate medians (black bars) and significance levels (*p*-values) with the Kruskal–Wallis test and Dunn’s post-correction. The total numbers of analyzed mice are given in parentheses.

We further assessed pro-inflammatory mediator secretion in MLN draining the infected intestines. *C. jejuni* infection was accompanied with elevated nitric oxide and TNF-α concentrations in placebo- and AC-treated mice (*p* < 0.01–0.001), but with lower levels in the latter versus the former (*p* < 0.05; Figure 6A,C). On day 6 following infection of mice from the placebo as opposed to the AC cohort, enhanced secretion of IL-6 and IFN-γ in MLN could be observed (*p* < 0.01 versus naive; Figure 6B,D). Hence, AC treatment could effectively dampen *C. jejuni*-induced pro-inflammatory immune responses in distinct parts of the intestinal tract.

### 3.5. Pro-Inflammatory Mediator Secretion in Extra-Intestinal Including Systemic Organs Following Treatment of Infected Mice with Activated Charcoal

**Figure 7:**
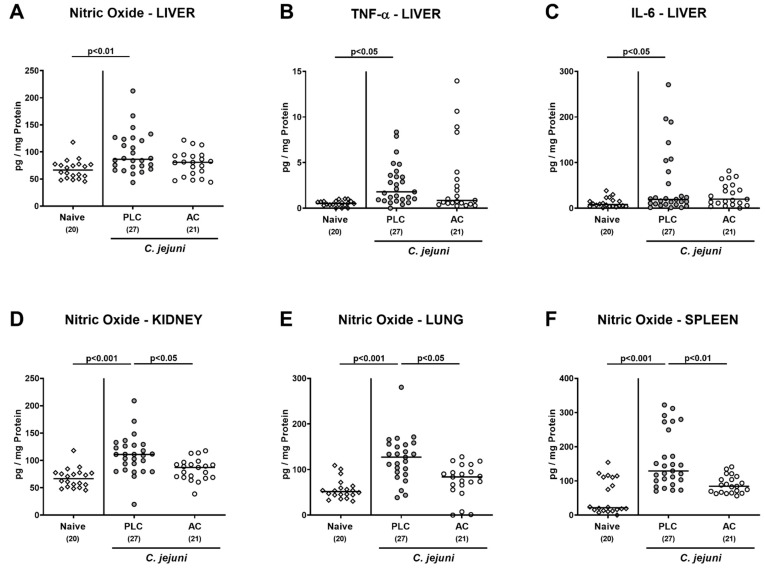
Pro-inflammatory mediator secretion in extra-intestinal organs following treatment of infected mice with activated charcoal. Starting on day (d) 2 post-infection (p.i), microbiota-depleted IL-10^−/−^ mice infected with *C. jejuni* strain 81–176 on d0 and d1 by gavage were perorally treated with either activated charcoal (AC; white circles) or placebo (PLC; gray circles) via the drinking water. On d6 p.i., extra-intestinal nitric oxide concentrations were measured in culture supernatants of ex vivo biopsies derived from (**A**) liver, (**D**) kidney, (**E**) lungs, and (**F**) spleen, whereas in addition, hepatic (**B**) TNF-α and (**C**) IL-6 secretion was assessed. Results from naive mice serving as non-infected, untreated controls were included for comparisons (white diamonds). Data pooled from four independent experiments were used to calculate medians (black bars) and significance levels (*p*-values) with the Kruskal–Wallis test and Dunn’s post-correction. The total numbers of analyzed mice are given in parentheses.

We were further interested whether the immune-modulatory effects of AC treatment were restricted to the intestinal tract of *C. jejuni*-infected mice or also effective beyond. Therefore, we assessed pro-inflammatory mediator secretion in extra-intestinal including systemic organs. On day 6 p.i., increased nitric oxide, TNF-α, and IL-6 concentrations were determined in liver samples taken from mice of the placebo (*p* < 0.05–0.01 versus naive), but not the AC cohort (Figure 7A–C), which also held true for higher nitric oxide concentrations measured in the kidneys, the lungs and the spleen of placebo as compared to AC-treated mice and to naive controls (*p* < 0.001 versus naive; *p* < 0.05–0.01 versus AC; Figure 7D–F).

**Figure 8:**
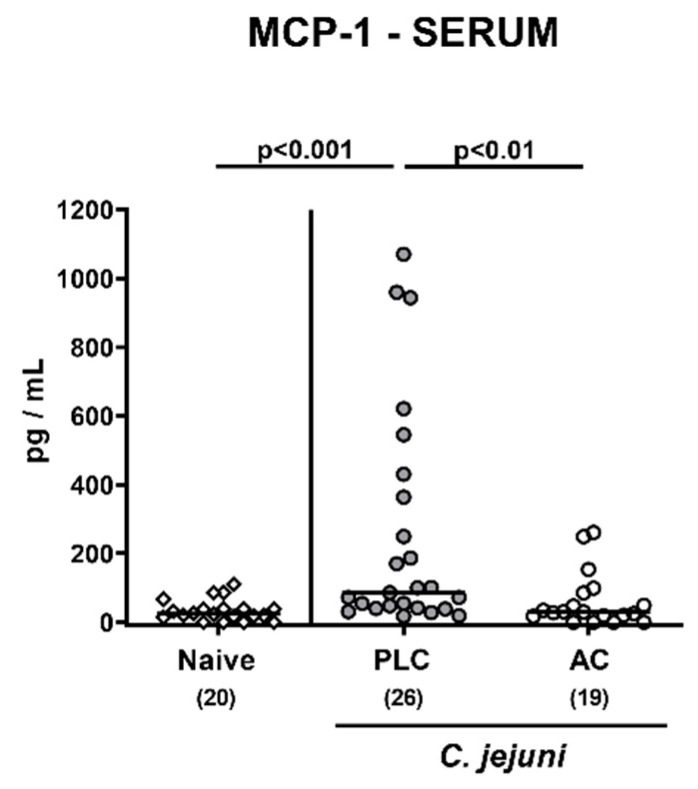
Systemic MCP-1 secretion following treatment of infected mice with activated charcoal. Microbiota-depleted IL-10^−/−^ mice were infected with *C. jejuni* strain 81–176 on day (d) 0 and d1 by gavage. Starting on d2 post-infection (p.i.), mice were perorally treated with either activated charcoal (AC; white circles) or placebo (PLC; gray circles) via the drinking water. On d6 p.i., systemic MCP-1 secretion was assessed in serum samples. Results from naive mice serving as non-infected, untreated controls were included for comparisons (white diamonds). Data pooled from four independent experiments were used to calculate medians (black bars) and significance levels (*p*-values) with the Kruskal–Wallis test and Dunn’s post-correction. The total numbers of analyzed mice are given in parentheses. Definite outliers were removed after being identified by the Grubb’s test (α = 0.001).

Furthermore, systemic MCP-1 secretion was enhanced upon placebo as opposed to AC treatment of *C. jejuni*-infected mice as assessed in serum samples taken on day 6 p.i. (*p* < 0.001 versus naive; Figure 8). Hence, AC treatment did not only dampen intestinal, but also extra-intestinal and remarkably, even systemic immune responses during acute murine campylobacteriosis.

## 4. Discussion

In traditional medicine charcoal-containing compounds and pure AC have been used for a long time as treatment options of diarrhea and enteric inflammation in a multitude of gastrointestinal morbidities and intoxications. Results from clinical studies further support the application of AC for alleviating enteric symptoms in irritable bowel syndrome and in the management of diarrhea caused by *Clostridoides difficile* toxins or in colorectal cancer, for instance [39]. Further microbiological investigations revealed that AC effectively inactivates bacterial enterotoxins such as cholera toxin by direct binding [48]. Despite these beneficial effects of AC in a broad range of gastrointestinal disorders, a potential use of AC in the treatment or prophylaxis of *C. jejuni*-induced enteritis has not been investigated so far. Our actual preclinical placebo-controlled evaluation of AC for the treatment of acute campylobacteriosis revealed that AC could effectively alleviate the severity of enteropathogenic disease. Following AC as compared to placebo treatment, microbiota-depleted IL-10^−/−^ mice (i) harbored up to 0.5 and 2.0 log orders of magnitude lower *C. jejuni* loads in the colon and ileum, respectively; (ii) were less distinctly suffering from diarrhea and wasting symptoms; (iii) displayed less distinct microscopic inflammatory (i.e., apoptotic) changes in the colonic epithelia, which were accompanied by (iv) lower immune cell numbers infiltrating the colonic mucosa and lamina propria, and remarkably, (v) by less pro-inflammatory mediator secretion as assessed in intestinal, extra-intestinal, and even systemic compartments.

Given the overall very high median *C. jejuni* loads of nearly 10^9^ CFU per gram luminal colon content, it is questionable whether the pronounced disease-alleviating effects observed upon AC application might be explained by the reduction in median pathogen numbers of approximately 0.5 log orders of magnitude in the distal large intestines and of 2.0 log orders in the terminal ileum alone. To date, the antibiotic effects of AC have been considered as rather negligible [49]. Nevertheless, it might be possible that AC treatment inhibits *C. jejuni* replication upon binding of distinct substrates that are required for growth of these fastidious microorganisms. While the corresponding mechanisms have not been investigated for *C. jejuni* to date, experimental data demonstrate that AC does, in fact, effectively inhibit the growth of *Escherichia coli* in rich liquid media by the binding of amino acids [50]. Given that amino acids are essential for maintaining *C. jejuni* metabolism [51], the resulting shortcoming in these molecules might additionally reduce the motility of *C. jejuni* in a similar manner as this was shown for *Proteus* species, for instance [52]. On the other hand, this putative antimicrobial effect of AC directed against *C. jejuni* is rather unlikely since it is known for decades that charcoal supplementation of growth media is successfully used to optimize culture of *C. jejuni* and other bacteria from the *Campylobacter* clade [53]. Furthermore, since AC was shown to inhibit intestinal colonization of *Klebsiella pneumoniae* in the course of antibiotic treatment it is tempting to speculate that AC might compromise the ability of enteropathogens to colonize and stably establish in the intestinal tract [54]. In case of *C. jejuni,* this feature of AC may strengthen the colonization resistance of mice and humans with a complex gut microbiota against the pathogen upon infection.

Importantly, AC has been proven to ameliorate diarrheal diseases by binding and adsorption of the causative agent to the surface and pores of the charcoal particles [38]. The ability of AC to adsorb and inactivate pathogenic bacteria by direct binding was proven experimentally for verotoxin-producing *Escherichia coli* [55]. In *C. jejuni* infection, AC may indirectly exert anti-inflammatory effects by binding and immobilization of the pathogens in the intestinal lumen which may result in the protection of the host from bacterial invasion. In consequence, AC blocks the activation of innate immune responses by LOS constituting a key mechanism of *C. jejuni*-induced immunopathology during acute campylobacteriosis. Given that the TLR-4 dependent signaling cascade induced by bacterial cell wall derived LOS is essential for *C. jejuni*-induced inflammation, a direct immuno-modulatory activity of AC in the amelioration of campylobacteriosis is further supported by its ability to directly inactivate bacterial LPS/LOS, and reactive oxygen radicals produced during inflammation as well as pro-inflammatory cytokines [40,53,56,57]. However, the mechanisms underlying the anti-pathogenic and anti-inflammatory effects of AC observed in the present study need to be substantiated by further investigations.

In view of the here obtained local and even systemic disease-alleviating effects of AC treatment during acute campylobacteriosis, it is hence highly likely that besides the rather modest pathogen-lowering effects, an orchestrated interplay of distinct immune-modulatory host responses might have contributed to the alleviated disease outcome in *C. jejuni*-infected mice. Clinically, AC-treated mice suffered from less severe diarrhea and, remarkably, also from less distinct wasting symptoms indicative for less severe systemic disease. When focusing onto the microscopic level, AC-treated mice exhibited less pronounced colonic epithelial cell apoptosis when compared to placebo counterparts, which was accompanied by lower abundance of innate cell populations such as macrophages and monocytes and of adaptive immune cell subsets including T- and B-cells. The AC-mediated dampening of the intestinal immune cell responses was accompanied by less distinct secretion of pro-inflammatory mediators such as nitric oxide, IL-6, TNF-α, or IFN-γ in intestinal compartments including the colon, ileum and MLN. Moreover, these anti-inflammatory effects of AC treatment were additionally observed in extra-intestinal tissues as indicated by lowered nitric oxide and IL-6 concentrations measured in liver, kidney, lungs, and strikingly, also systemically given nitric oxide and MCP-1 concentrations in spleen and serum samples, respectively, that did not differ from basal levels when taken from AC as opposed to placebo-treated mice on day 6 p.i. To date, information on the clinical effects of AC in experimental in vivo models of intestinal inflammation are scarce, however. One study could demonstrate the anti-inflammatory effects of AC given that oral application of an AC containing formulation termed Le Carbone ameliorated disease in mice suffering from chemically induced acute colitis [58]. The pronounced anti-inflammatory including anti-apoptotic effects of Le Carbone were attributed to a down-regulation of Signal transducer and activator of transcription 3 (STAT-3), nuclear factor kappa-light-chain-enhancer of activated B cells (NF-κB), TNF-α, IL-1 β, and caspases [58].

Given the relatively short treatment period of four days (i.e., from day 2 until day 6 p.i.), the observed immune-modulatory effects are even more remarkable. One could hypothesize that the health-beneficial effects might be even more pronounced upon initiation of oral AC application before infection. This will be addressed in our future studies. Regarding a transfer of the clinical data obtained in the murine infection model to the treatment of campylobacteriosis in humans, it is of particular importance that induction of intestinal inflammation and apoptosis by LOS and ROS are pivotal for induction and progression of human campylobacteriosis [20]. Given that the severity of the pathogen-induced enteritis symptoms is directly linked to post-infectious sequelae, as shown in an earlier clinical study [12], it is tempting to speculate that the amelioration of acute campylobacteriosis by AC treatment may additionally reduce the risk for the development of autoimmune diseases including Guillain–Barré syndrome and reactive arthritis in *C. jejuni*-infected patients, for instance. The promising application of AC in the management of *C. jejuni* infection is further supported by the fact that the drug can be regarded as rather non-toxic and is available over the counter worldwide. It is further important to note that the short-term use of AC in acute campylobacteriosis and other diarrheal diseases avoids unwanted side effects of AC such as inactivation of other medications and the blockage of essential vitamin uptake, for instance. One needs to take further into consideration, that given the worldwide threat of antimicrobial resistances, antibiotics-independent approaches to combat (entero)pathogenic including *Campylobacter* infections are becoming more and more important.

## 5. Conclusions

The results of our preclinical placebo-controlled intervention study provide evidence for the first time that AC exhibits anti-pathogenic and immune-modulatory properties in acute *C. jejuni* infection. Given that AC exerts diverse effects such as inactivation of live pathogens, of endotoxins, and of pro-inflammatory mediators, the substance may be considered as a potent and safe short-term treatment option for acute campylobacteriosis in humans. The results obtained here complete our knowledge on natural compounds as promising antibiotic-independent options to combat campylobacteriosis and to provide infected patients with valid non-toxic alternatives to ameliorate the symptoms of this frequent and often painful disease.

## Data Availability

The corresponding author provides the data from this study on request.

## Supplementary Materials

The following are available online at https://www.mdpi.com/article/10.3390/microorganisms9071424/s1, Figure S1: Kinetic survey of diarrheal symptoms following treatment of infected mice with activated charcoal.
