# Supplementary material for: Disease-Alleviating Effects of Peroral Activated Charcoal Treatment in Acute Murine Campylobacteriosis
Source: Microorganisms. 2021 Jun 30;9(7):1424. doi: 10.3390/microorganisms9071424 (PMC8307340; doi:10.3390/microorganisms9071424)
Supplement: Supplementary file 1 [file microorganisms-09-01424-s001.zip › microorganisms-1267643-SI.pdf]

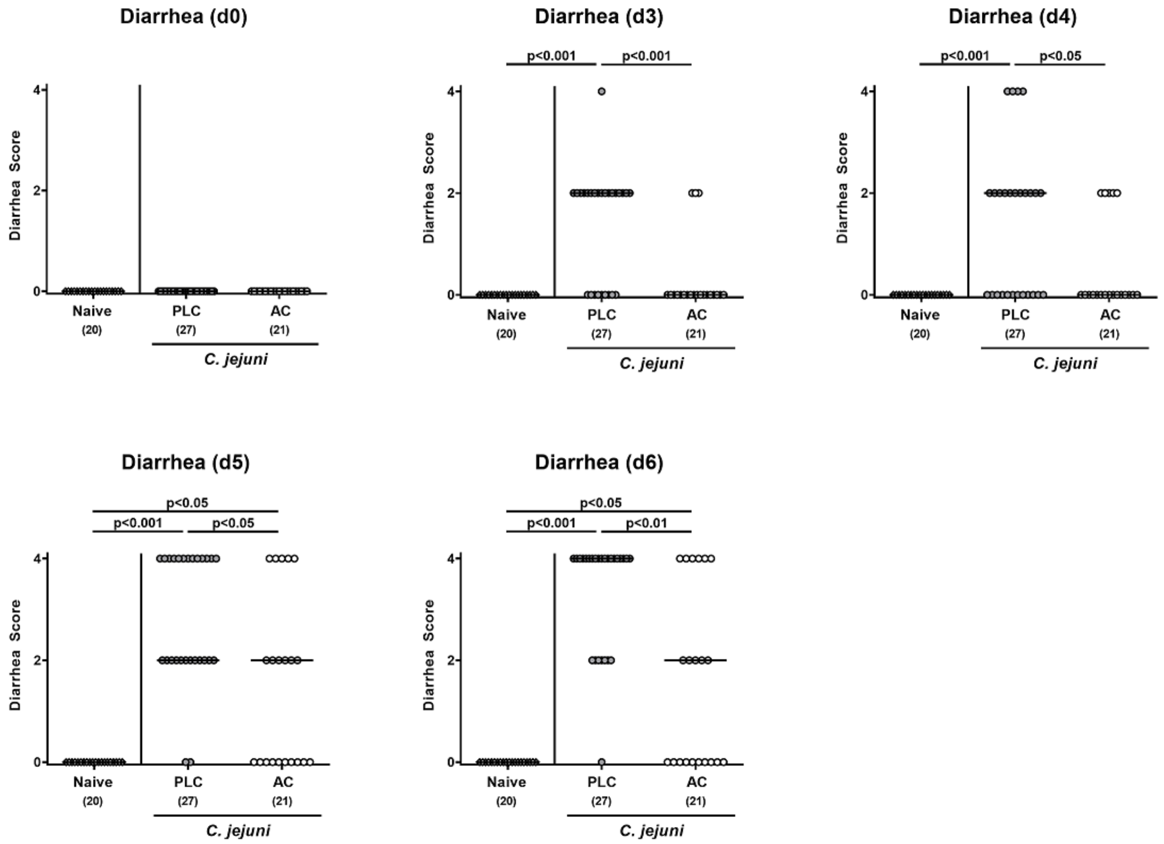

Figure S1: Kinetic survey of diarrheal symptoms following treatment of infected mice with activated charcoal.
